# The Hospital Secretariat

**Published:** 1906-12-08

**Authors:** 


### THE HOSPITAL SECRETARIAT.

#### The Late Thomas Beattie Campbell.

The death has occurred at his residence in Sutton of the late Secretary of the Royal Westminster Ophthalmic Hospital, Mr. Thomas Beattie Campbell, at the age of 57. Mr. Campbell was a grandnephew of the poet Thomas Campbell, and was also related to Mr. Beattie, Surgeon to George III. He had led a somewhat chequered life, having wandered through the United States and held an appointment on the Grand Trunk Railway of Canada. In 1883 he was appointed Secretary to the Royal Westminster Ophthalmic Hospital, which post he filled until stricken down by paralysis when spending his Easter holiday in Lisbon in 1895. Mr. Campbell was awarded a pension by the Committee, but has not lived long to receive it.

#### Mr. C. L. Todd.

St. George's Hospital will seem strange and odd to many governors when Mr. Todd finally retires from the post of superintendent and secretary, after forty-four years' continuous service in the hospital.

Mr. Todd has ever made the welfare of the hospital his first consideration. The governors in affirming this fact have declared with justice that Mr. Todd has devoted himself, in the most indefatigable and enthusiastic manner, to the maintenance of the interests and reputation of St. George's Hospital. One of the most courteous, calm, and resourceful of secretaries, Mr. Todd's personal popularity has been so great as to be almost unique in the public service. We have not heard that Mr. Todd is to have, as he ought to have, a pension granted to him by the governors on his retirement from the service. We welcome the movement which is on foot to present Mr. Todd with a testimonial as some mark of the esteem in which he is held, but justice demands, that, any such movement shall, in no way, militate against a pension. It ought in fact to make in favour of the most generous consideration, when the amount of this pension comes up to be fixed by the governors. It is perfectly clear, that, in these times, no great hospital can afford to take forty-four years' service, such as Mr. Todd has rendered, and not grant a pension, when the day of retirement arrives. We wish Mr. Todd the best of health and happiness for the rest of his life. There should be no "winter of discontent" in Mr. Todd's case, for a life of personal service for the sick should mean joy and contentment in the peaceful days of retirement and rest.

